# Supplementary material for: Control of intracellular pH and bicarbonate by CO2 diffusion into human sperm
Source: Nat Commun. 2023 Sep 5;14:5395. doi: 10.1038/s41467-023-40855-0 (PMC10480191; doi:10.1038/s41467-023-40855-0)
Supplement: Supplementary file 1 — Supplementary Information [file 41467_2023_40855_MOESM1_ESM.pdf]

Supplementary Information

Control of intracellular pH and bicarbonate by CO_2 diffusion into human sperm

Elena Grahn[1§], Svenja V. Kaufmann[2§], Malika Askarova[1], Momchil Ninov[2,3], Luisa M. Welp[2,3], Thomas K. Berger[1,4], Henning Urlaub[2,3], and U. Benjamin Kaupp[1,5]

[1]Max Planck Institute for Neurobiology of Behavior–caesar, Molecular Sensory Systems, Ludwig-Erhard-Allee 2, 53175 Bonn, Germany; [2]Max Planck Institute for Multidisciplinary Sciences, Bioanalytical Mass Spectrometry, Am Fassberg 11, 37077 Göttingen, Germany; [3]University Medical Center Göttingen, Institute of Clinical Chemistry, Bioanalytics, Robert-Koch-Strasse 40, 37075 Göttingen, Germany; [4]Department of Neurophysiology, Institute of Physiology and Pathophysiology, Philipps-University Marburg, Deutschhausstrasse 1-2, 35037 Marburg, Germany; [5]Life & Medical Sciences Institute (LIMES), University Bonn, Carl-Troll-Strasse 31, 53115 Bonn, Germany

[§]E.G. and S.V.K. contributed equally to this work.

Send correspondence to:

Thomas K. Berger
Department of Neurophysiology, Institute of Physiology and Pathophysiology, Philipps-University Marburg
Deutschhausstrasse 1-2
35037 Marburg, Germany.
Tel.: ++496421-28-62398, Fax: ++496421-28-62306
e-mail: thomas.berger@uni-marburg.de

U. Benjamin Kaupp
LIMES Institute, University Bonn
Carl-Troll-Strasse 31
53175 Bonn, Germany
Tel.: ++49228-9656-100, Fax: ++49228-9656-9273
e-mail: ubkaupp@uni-bonn.de

Henning Urlaub
Max-Planck-Institute for Multidisciplinary Sciences
Bioanalytical Mass Spectrometry Group
Am Fassberg 11
37077 Göttingen, Germany
Tel.: ++49551 201 1060 (Office)/-1500 (Lab)
email: henning.urlaub@mpinat.mpg.de

Supplementary Note 1. Summary of reports on alkalization during capacitation.

Several reports have been cited for sperm alkalization during capacitating conditions (Supplementary Table 6) [1-11]. Meizel & Deamer studied acrosomal rather than cytosolic pH; changes in pH_i during capacitation were not examined[1]. Babcock and colleagues studied pH_i increase by NH_4Cl or changes in $[K^+]$; neither capacitation was studied nor HCO_3^- was employed or mentioned[2]. Parrish and colleagues studied the effect of heparin on alkalization[3]. Both pH_o and pH_i increased during 5 h incubation. The mechanism by which heparin changes pH_i is not known. Zeng and colleagues report an increase of $\Delta pH_i = 0.2$ after HCO_3^- addition[4]; spontaneous recovery from alkalization does not involve Na^+/H^+ exchange or other ion-dependent transport pathways. Vredenburgh-Wilberg & Parrish observed ΔpH_i of 0.2 upon heparin treatment for 5 h (ref. 5). Nakanishi and colleagues studied acrosomal pH_i during capacitation[6]. Demarco and colleagues measured $\Delta pH_i = 0.15$ upon addition of 5 mM HCO_3^- (ref. 7). Finally, Carlson and colleagues did not observe a pH_i change upon HCO_3^- treatment[11]. In summary, three out of nine studies reported ΔpH_i of 0.1-0.2 upon capacitation; one reported a larger increase ($\Delta pH_i = 0.75$) that was related to CFTR activity[8]; one study reported no change in pH_i (ref. 11). We suggest that during lengthy incubations in an open system, CO_2 leaves the solution and, thereby, shifts the $CO_2/HCO_3^-/H^+$ equilibrium to the left, and the solution alkalizes.

Supplementary Note 2. Kinetics of CO_2 diffusion across membranes.

We calculated the kinetics of CO_2 diffusion across cell membranes. The mechanisms of CO_2/HCO_3^- transport are controversial[12-14], but for the following estimate irrelevant. If only CO_2 diffusion contributes, $\tau = V/(A\ P_{CO2})$, wherein V is the cell volume, A is the membrane surface, and P_{CO2} is the membrane permeability. Values for P_{CO2} vary between $1.5x10^{-3}$ cm s^{-1} and 0.33 cm s^{-1} (ref 14). Taking the smallest P_{CO2}, for a flagellum (radius r = 0.125 µm; length l = 50 µm) and a spherical CHO cell (r = 10 µm), τ becomes 25 ms and 333 ms, respectively. Larger P_{CO2} values yield even faster transport rates. Thus, the kinetics of cell acidification are not rate-limited by CO_2 diffusion across the membrane. Rather, the rate constants of the $CO_2 + H_2O \rightarrow HCO_3^- + H^+$ reaction determine the rate of k the changes in pH_i. In the absence of carbonic anhydrase (CA), k = 0.037 s^{-1} or τ = 27 s (ref. 15). In conclusion, the large difference of τ_{off} of ΔpH_i between human sperm, CHO cells, and HEK293 cells must be caused by different CA inventories.

Supplementary References

1 Meizel, S. & Deamer, D. W. The pH of the hamster sperm acrosome. *J. Histochem. Cytochem.* **26**, 98-105 (1978).

2 Babcock, D. F., Rufo, G. A., Jr. & Lardy, H. A. Potassium-dependent increases in cytosolic pH stimulate metabolism and motility of mammalian sperm. *Proc. Natl. Acad. Sci. USA* **80**, 1327-1331 (1983).

3 Parrish, J. J., Susko-Parrish, J. L. & First, N. L. Capacitation of bovine sperm by heparin: inhibitory effect of glucose and role of intracellular pH. *Biol. Reprod.* **41**, 683-699 (1989).

4 Zeng, Y., Oberdorf, J. A. & Florman, H. M. pH regulation in mouse sperm: identification of Na^+-, Cl^--, and HCO_3^--dependent and arylaminobenzoate-dependent regulatory mechanisms and characterization of their roles in sperm capacitation. *Dev. Biol.* **173**, 510-520 (1996).

5 Vredenburgh-Wilberg, W. L. & Parrish, J. J. Intracellular pH of bovine sperm increases during capacitation. *Mol. Reprod. Dev.* **40**, 490-502 (1995).

6 Nakanishi, T., Ikawa, M., Yamada, S., Toshimori, K. & Okabe, M. Alkalinization of acrosome measured by GFP as a pH indicator and its relation to sperm capacitation. *Dev. Biol.* **237**, 222-231 (2001).

7 Demarco, I. A. *et al.* Involvement of a Na^+/HCO_3^- cotransporter in mouse sperm capacitation. *J. Biol. Chem.* **278**, 7001-7009 (2003).

8 Xu, W. M. *et al.* Cystic fibrosis transmembrane conductance regulator is vital to sperm fertilizing capacity and male fertility. *Proc. Natl. Acad. Sci. USA* **104**, 9816-9821 (2007).

9 Cross, N. L. & Razy-Faulkner, P. Control of human sperm intracellular pH by cholesterol and its relationship to the response of the acrosome to progesterone. *Biol. Reprod.* **56**, 1169-1174 (1997).

10 Lopez-Gonzalez, I. *et al.* Membrane hyperpolarization during human sperm capacitation. *Mol. Hum. Reprod.* **20**, 619-629 (2014).

11 Carlson, A. E., Hille, B. & Babcock, D. F. External Ca^{2+} acts upstream of adenylyl cyclase SACY in the bicarbonate signaled activation of sperm motility. *Dev. Biol.* **312**, 183-192 (2007).

12 Missner, A. & Pohl, P. 110 years of the Meyer-Overton rule: predicting membrane permeability of gases and other small compounds. *Chem. Phys. Chem.* **10**, 1405-1414 (2009).

13 Boron, W. F., Endeward, V., Gros, G., Musa-Aziz, R. & Pohl, P. Intrinsic CO_2 permeability of cell membranes and potential biological relevance of CO_2 channels. *Chem. Phys. Chem.* **12**, 1017-1019 (2011).

14 Endeward, V., Arias-Hidalgo, M., Al-Samir, S. & Gros, G. CO_2 Permeability of Biological Membranes and Role of CO_2 Channels. *Membranes (Basel)* **7** (2017).

15 Roughton, F. J. W. The Kinetics and Rapid Thermochemistry of Carbonic Acid. *J. Am. Chem. Soc.* **11**, 2930-2934 (1941).

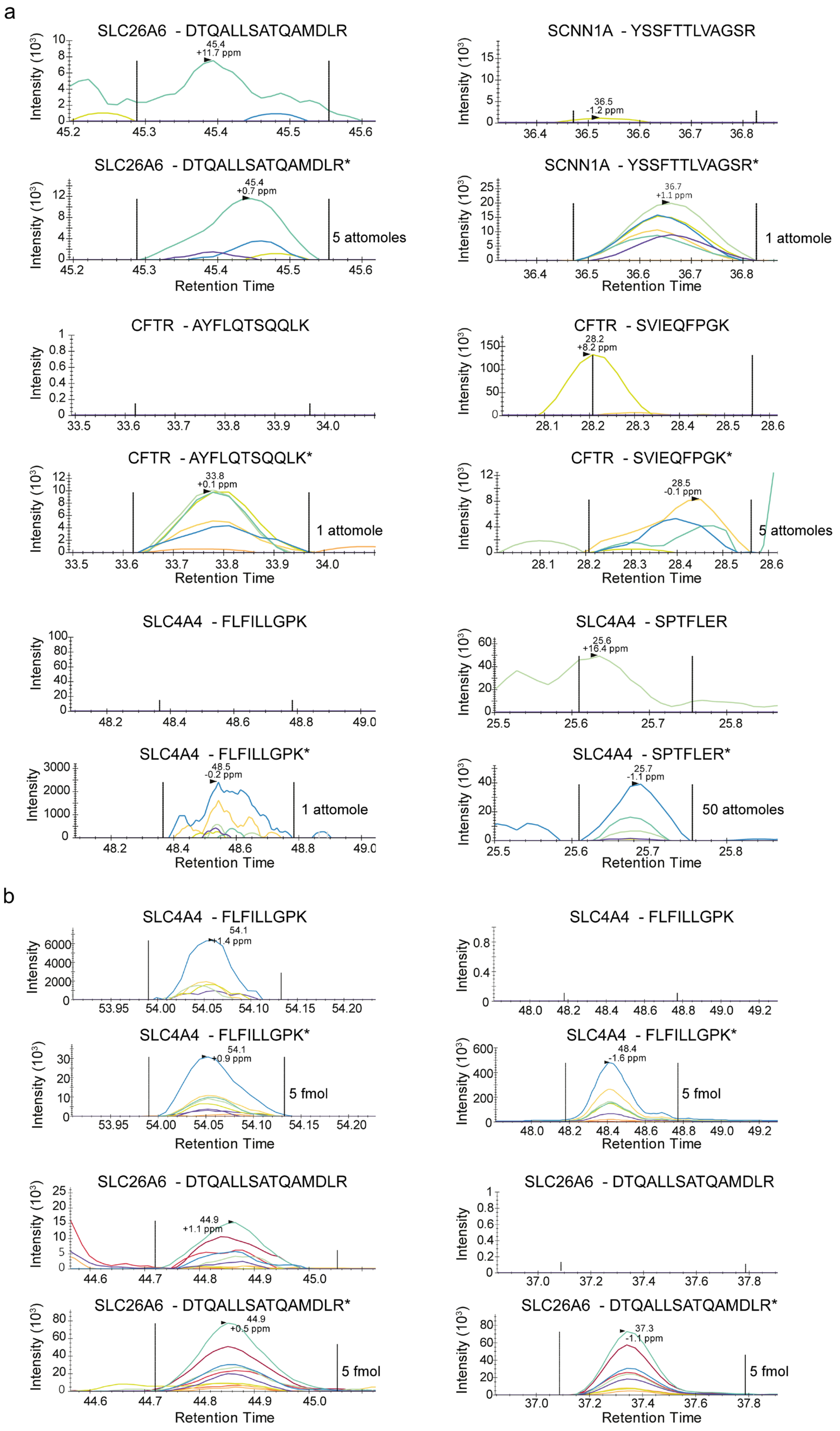
a
SLC26A6 - DTQALLSATQAMDLR
45.4
+11.7 ppm
SCNN1A - YSSFTTLVAGSR
36.5
-1.2 ppm
SLC26A6 - DTQALLSATQAMDLR*
45.4
+0.7 ppm
5 attomoles
SCNN1A - YSSFTTLVAGSR*
36.7
+1.1 ppm
1 attomole
CFTR - AYFLQTSQQLK
CFTR - SVIEQFPGK
28.2
+8.2 ppm
CFTR - AYFLQTSQQLK*
33.8
+0.1 ppm
1 attomole
CFTR - SVIEQFPGK*
28.5
-0.1 ppm
5 attomoles
SLC4A4 - FLFILLGPK
SLC4A4 - SPTFLER
25.6
+16.4 ppm
SLC4A4 - FLFILLGPK*
48.5
-0.2 ppm
1 attomole
SLC4A4 - SPTFLER*
25.7
-1.1 ppm
50 attomoles
b
SLC4A4 - FLFILLGPK
54.1
+1.4 ppm
SLC4A4 - FLFILLGPK
SLC4A4 - FLFILLGPK*
54.1
+0.9 ppm
5 fmol
SLC4A4 - FLFILLGPK*
48.4
-1.6 ppm
5 fmol
SLC26A6 - DTQALLSATQAMDLR
44.9
+1.1 ppm
SLC26A6 - DTQALLSATQAMDLR
SLC26A6 - DTQALLSATQAMDLR*
44.9
+0.5 ppm
5 fmol
SLC26A6 - DTQALLSATQAMDLR*
37.3
-1.1 ppm
5 fmol
Intensity (10³)
Intensity
Retention Time

Supplementary Figure 1. MS signal of lowest LOD values and positive controls.
(a) Transition channels of the most sensitive PRM analysis of all used peptides corresponding to SCNN1A, CFTR, SLC4A4, and SLC26A6. Peptide sequences are given; asterisks denote the ^{13}C- and ^{15}N-labelled amino acids in standard peptides. Upper panels: No specific PRM signals detected for endogenous peptides derived from 90,000 sperm cells; lower panels: PRM signals detected from 1 attomole (5 attomoles for DTQALLSATQAMDLR (SLC26A6) and 50 attomoles for SPTFLER (SLC4A4)) of standard peptide spiked into endogenous peptides derived from 90,000 sperm cells. Transitions of the different fragment ions are color-coded. **(b)** Transition channels of PRM analysis of heavy peptides spiked into HEK293 cell peptides as positive control. Upper panel: On the left, 5 fmol of standard peptide FLFILLGPK (SLC4A4) spiked into 1 µg of HEK293 cell peptides, signal for standard and endogenous peptide can be detected; on the right, 5 fmol of this standard peptide measured without matrix, no endogenous signal can be detected. Lower panel: On the left, 5 fmol of standard peptide DTQALLSATQAMDLR (SLC26A6) spiked into 1 µg of HEK293 peptides, signal for standard and endogenous signal can be detected; on the right, 5 fmol of this peptide measured without matrix, no endogenous signal can be detected.

a Calibration curves for quantification of carbonic anhydrase 2.

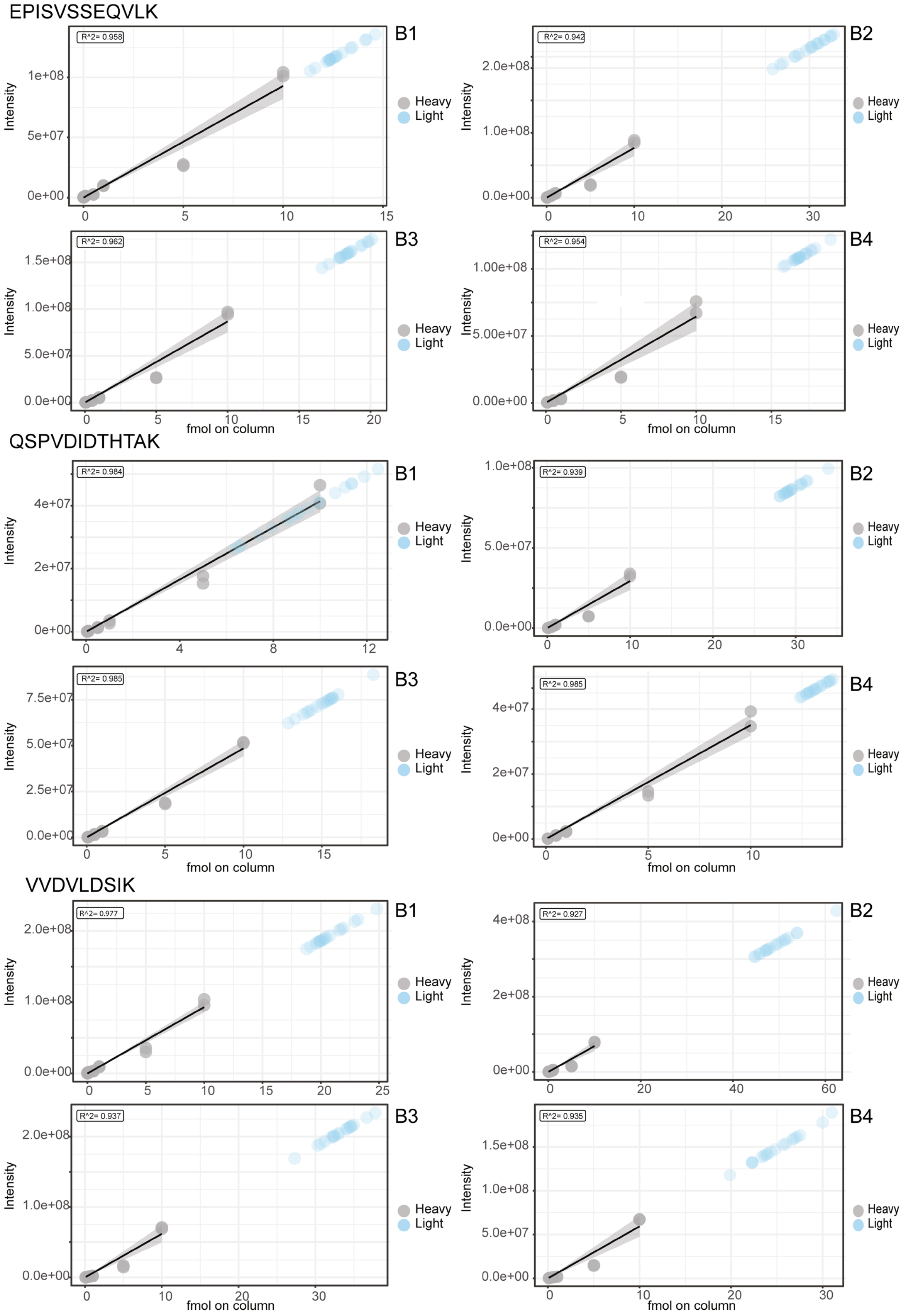

b Calibration curves for quantification of chloride anion exchanger SLC26A3.

LIDAVGFSPLR

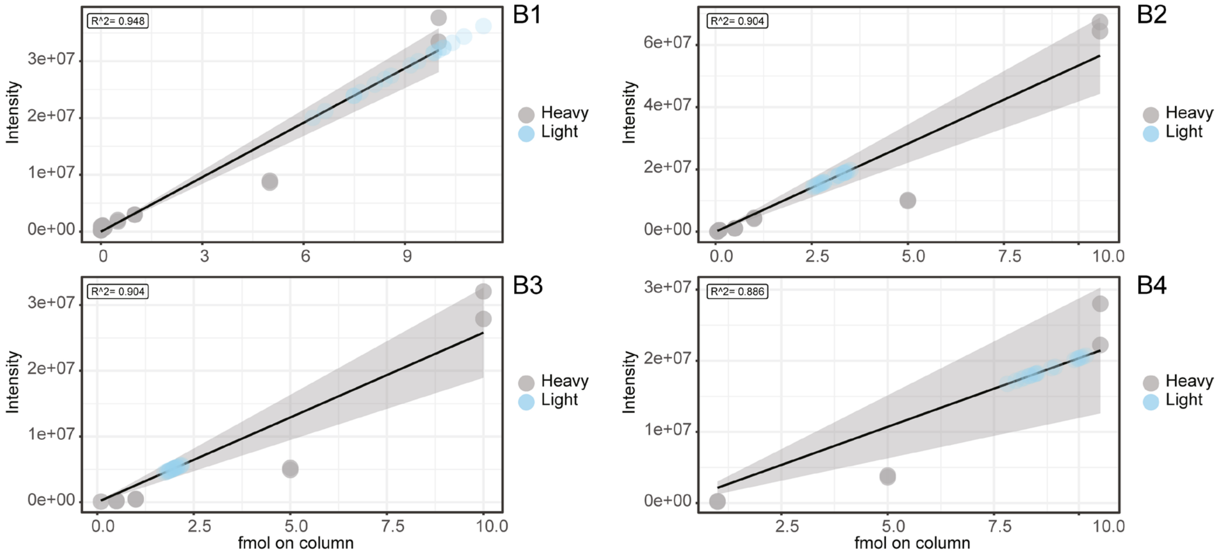

YEFFDGEVK

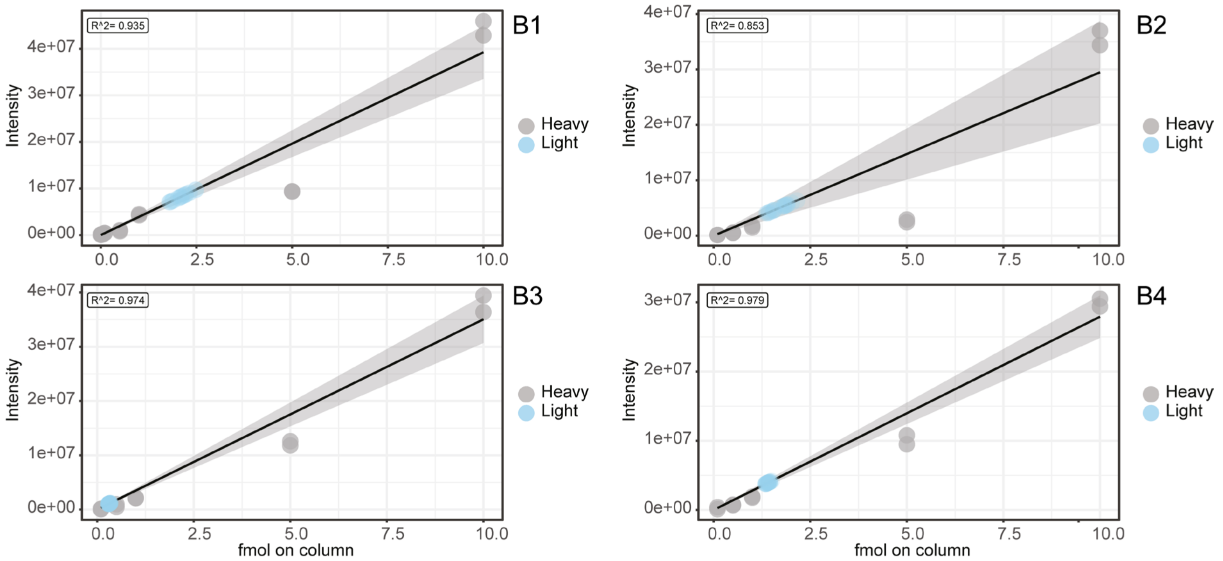

c Calibration curves for quantification of CatSper1.

SLIHDAPGPAASR

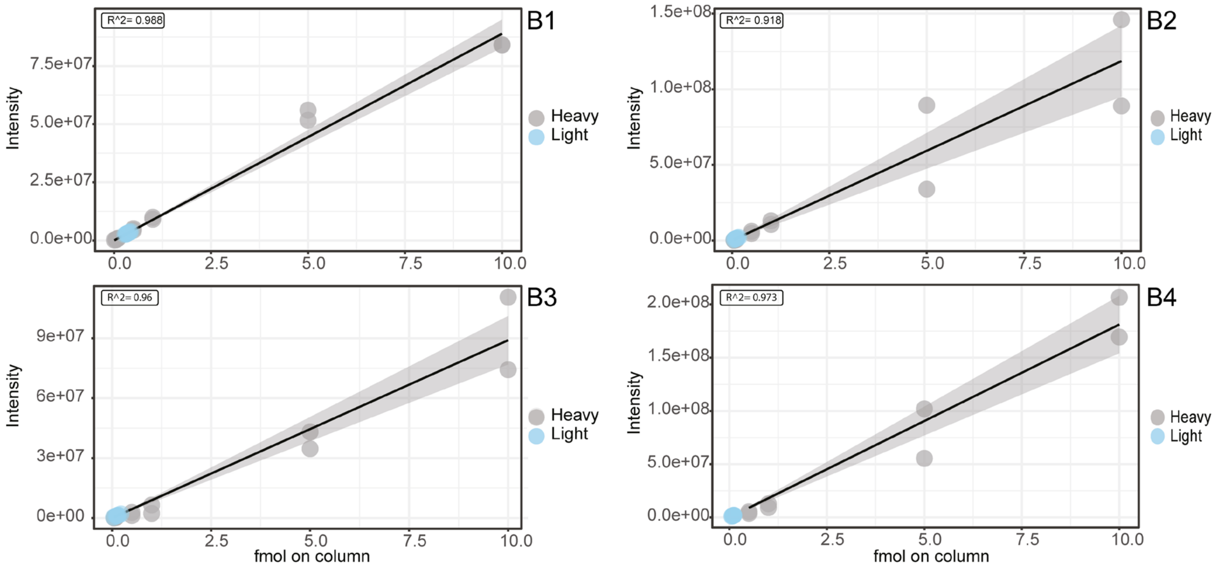

SYGEDYHDELQR

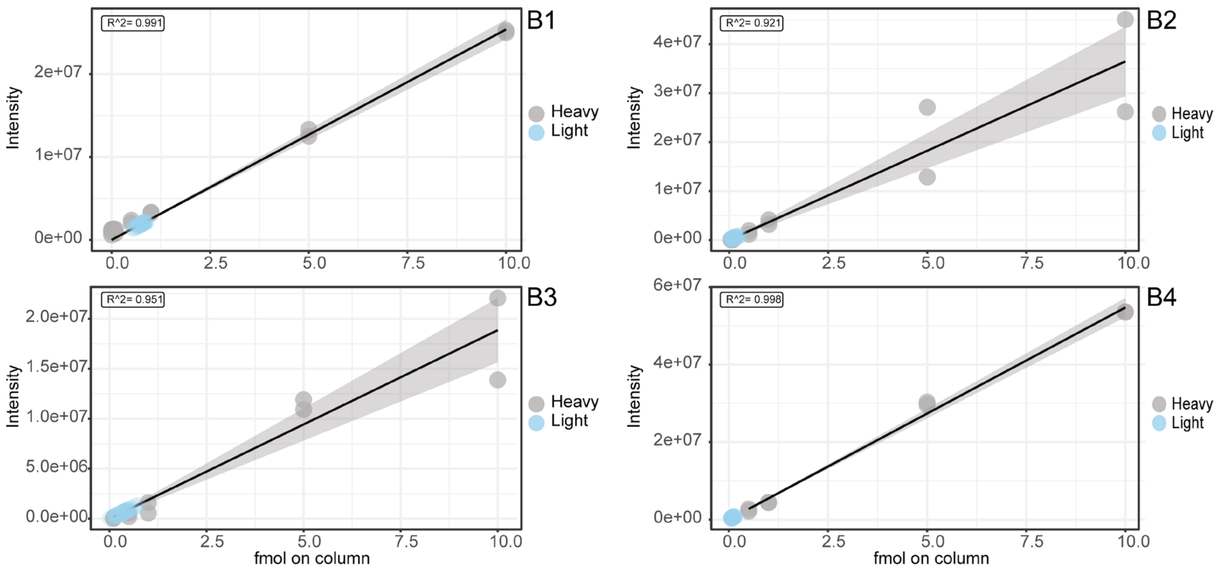

d Calibration curves for quantification of CatSper2.

LQYNLEER

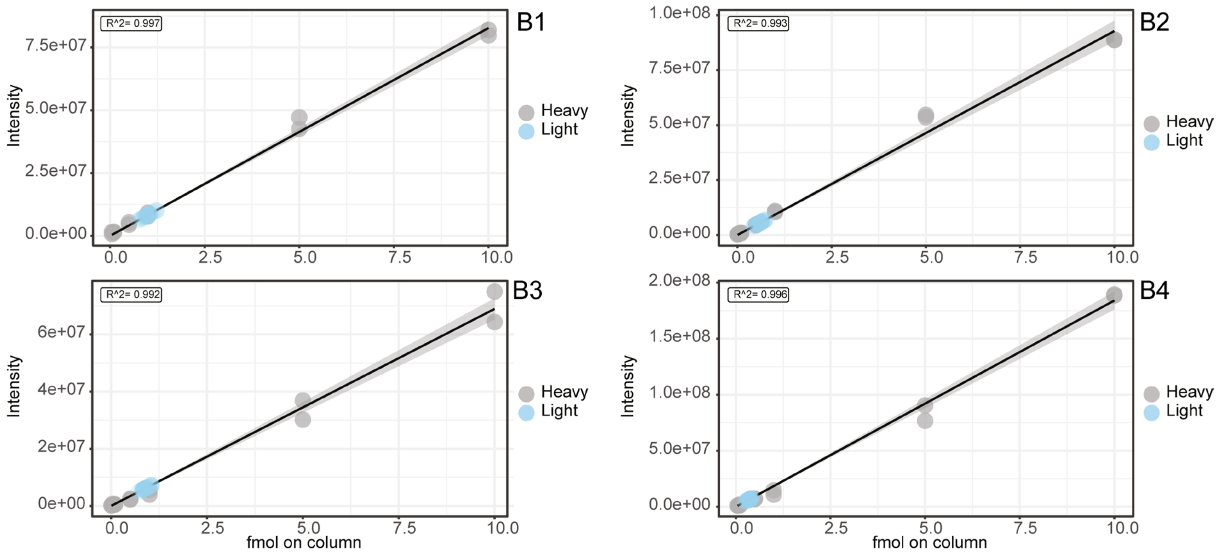

e Calibration curves for quantification of CatSper3.

LIGYSQGIR

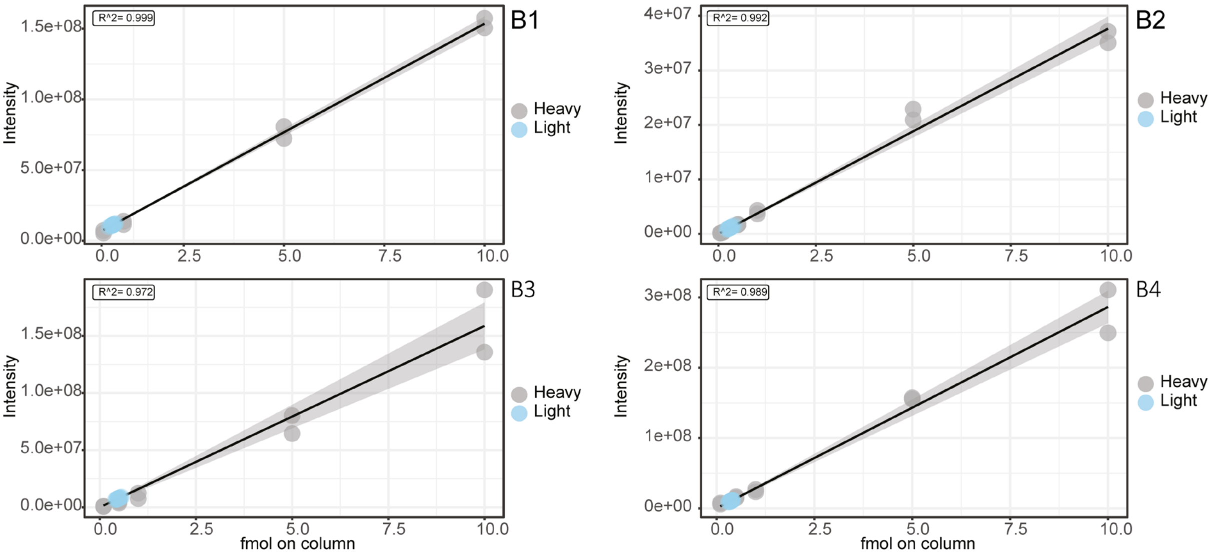

TVASVLLR

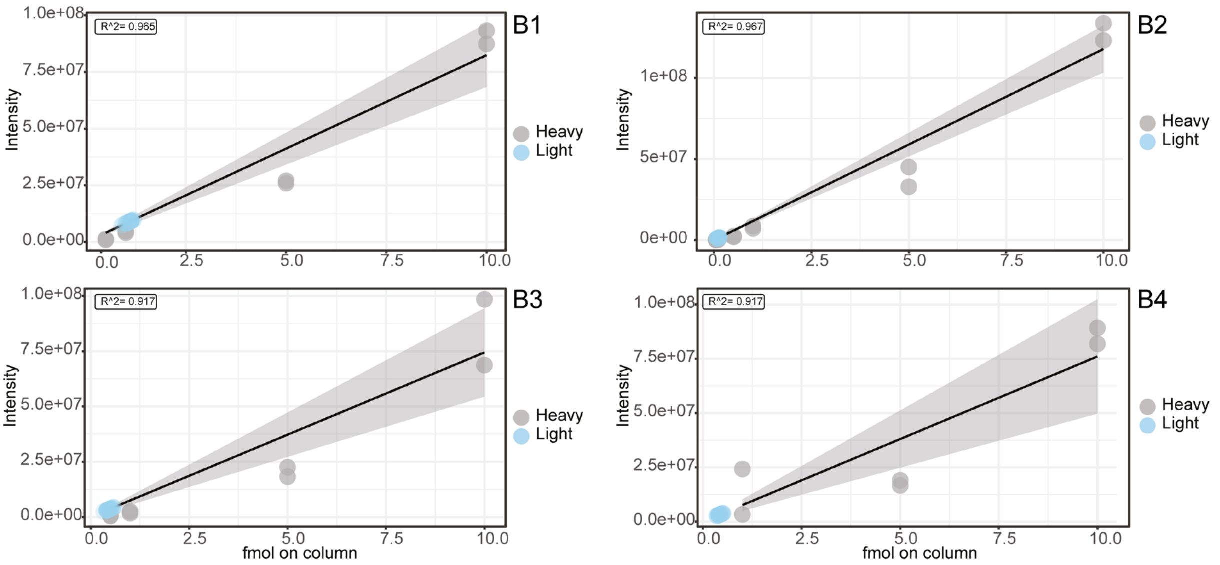

f Calibration curves for quantification of CatSper4.

FNQEQESEVLNR

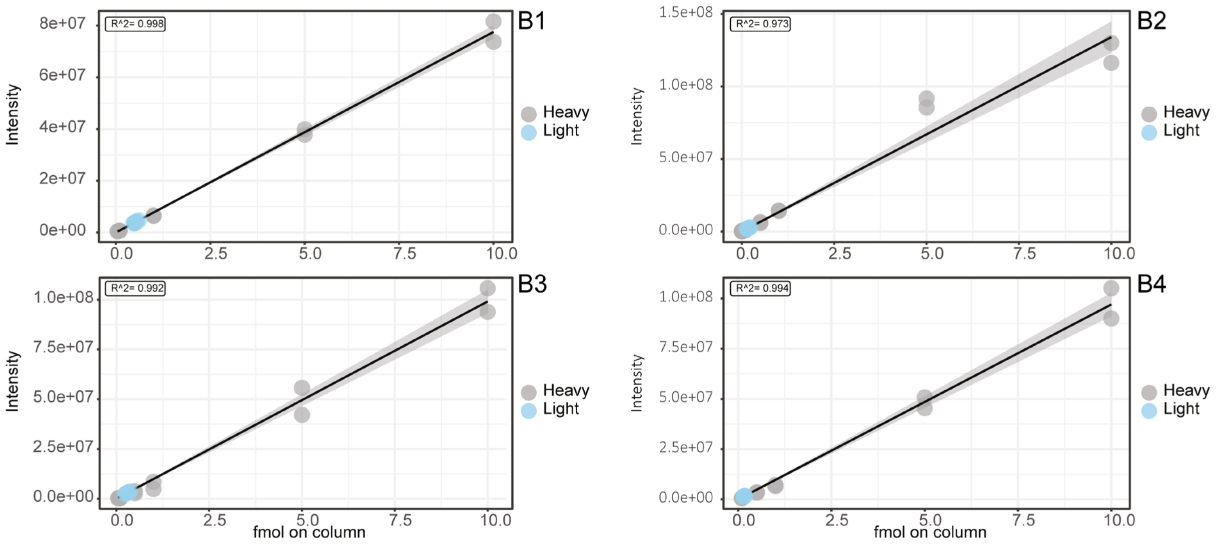

VHDSSSQILLK

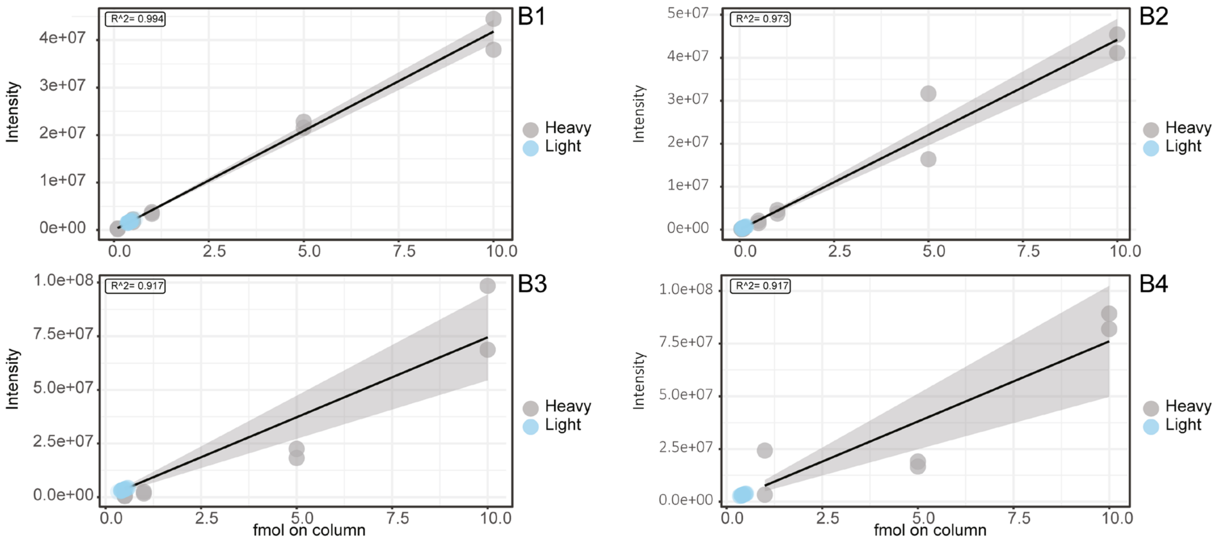

g Calibration curves for quantification of CatSperB.

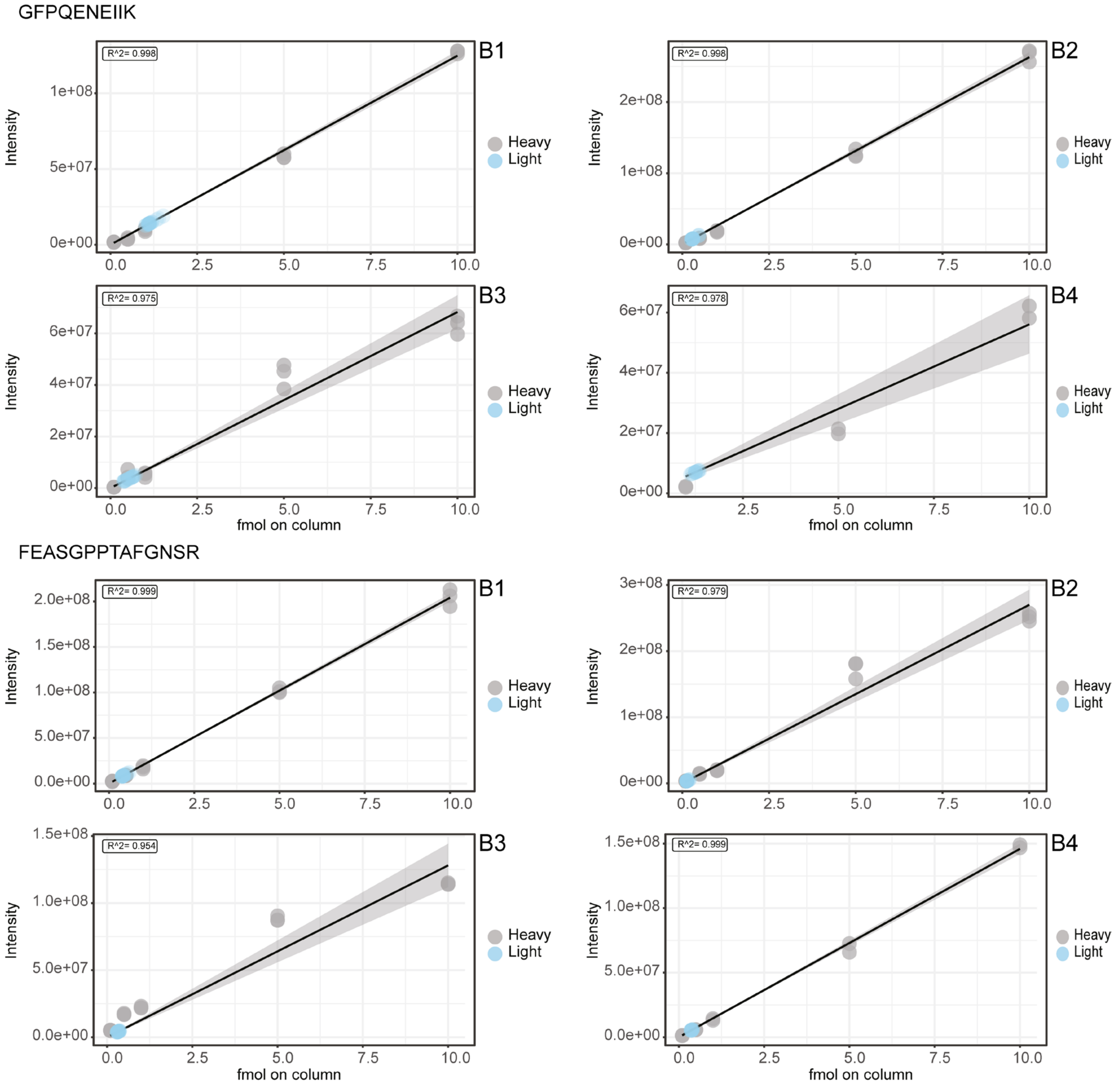

h Calibration curves for quantification of Slo3.

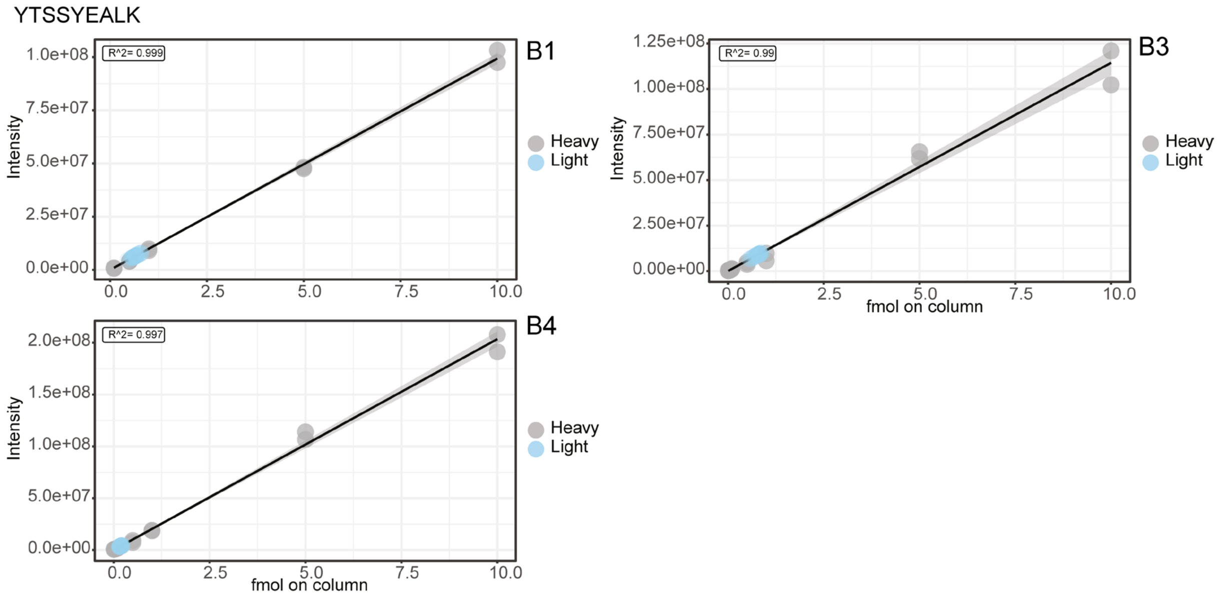

Supplementary Figure 2. Calibration curves for all quantified proteins.

(a-h) Calibration curves generated for quantification of CA2, SLC26A3, CatSper1-4, CatSperB, and Slo3. Calibration curves from all biological replicates and for all peptides of the respective proteins are shown. X-axis shows fmol of peptide on column and y-axis shows the signal intensity. Grey data points correspond to signal generated by standard peptide, turquoise data points correspond to the signals generated by endogenous peptides. R^2 value for each calibration curve is given in the inset. Shaded grey area indicates confidence interval of the linear regression. Source data is provided as a source data file.

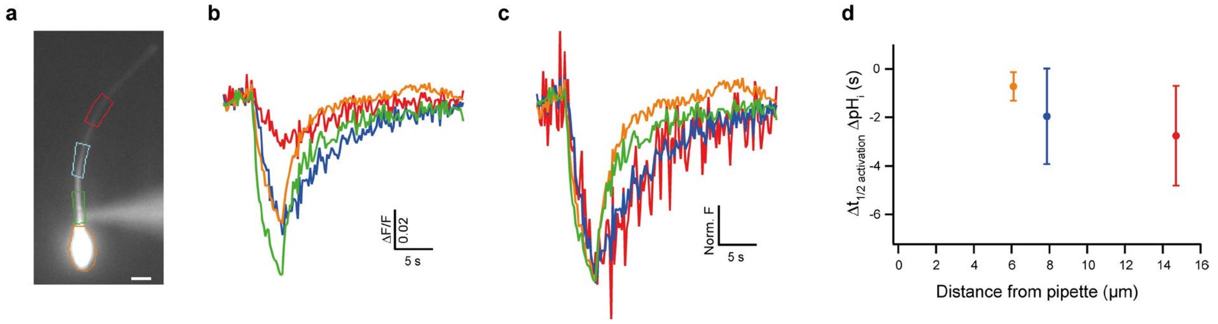

Supplementary Figure 3. PCF recordings of human sperm. Comparison of changes in pH_i in different compartments of the cell.

(**a**) pHrodo-Red-loaded human sperm with attached patch pipette and manually chosen ROIs. Scale bar 3 µm. (**b**) Alkalization induced by a +70-mV depolarization step and subsequent H_v1 activation during PCF recording in presence of CO_2/HCO_3^- (5%/25 mM). Superposition of fluorescent signal of different ROIs. (**c**) Normalized superposition of fluorescent signals from (b). (**d**) Difference of $t_{1/2}$ of H_v1-induced alkalization activation from respective ROIs compared to the ROI at the pipette (green, n_{cells} = 3, data points and error bars show mean ± SD). Source data is provided as a source data file.

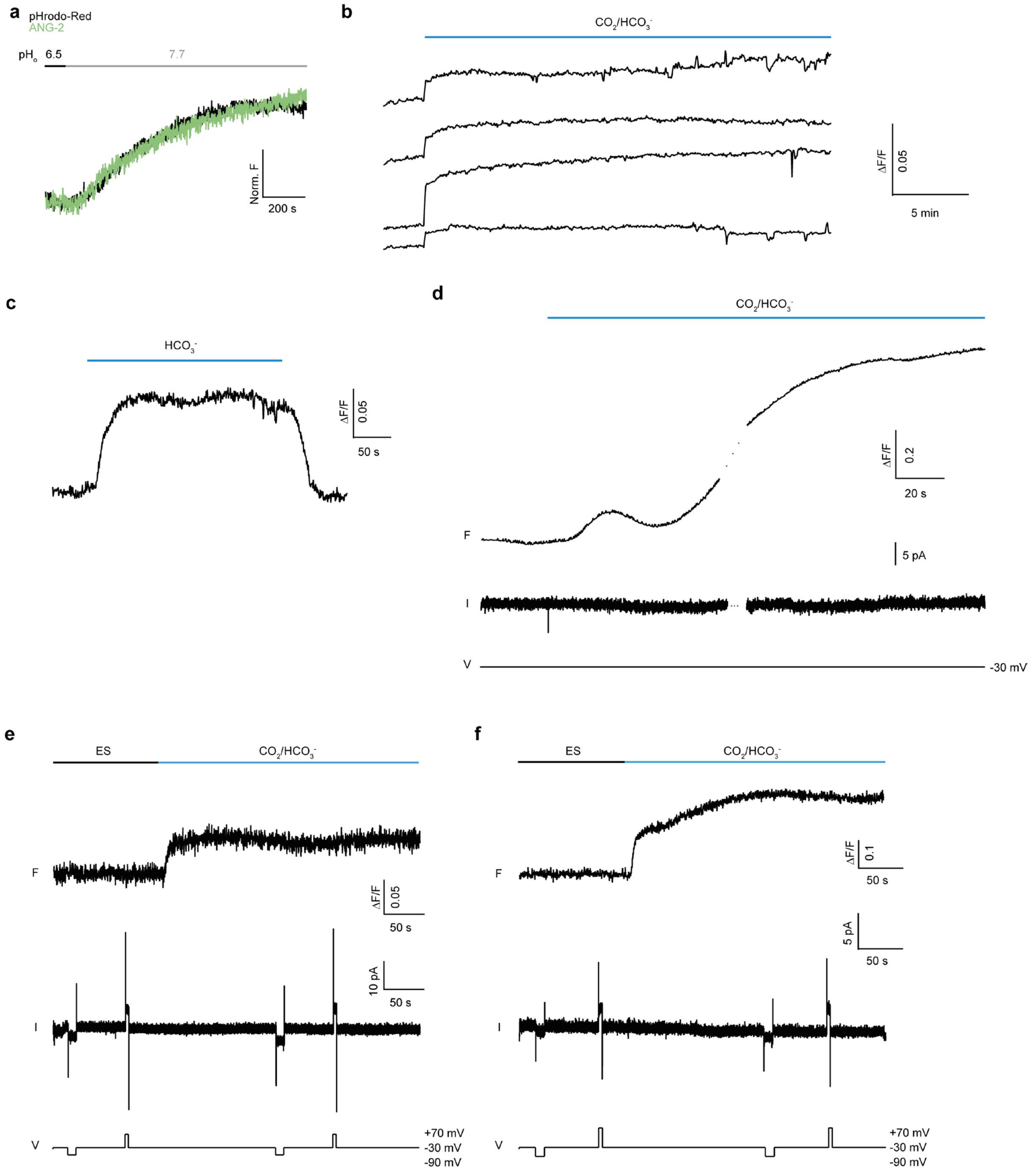

Supplementary Figure 4. Additional experiments testing for Na^+/H^+ exchange and behavior of sperm exposed to CO_2/HCO_3^-.

(a) Superposition of the fluorescence change of pHrodo-Red (black) and ANG-2 (green) in a sperm cell upon switching from pH_o6.5 to pH_o7.7. **(b)** Long-term recording of pH_i with pHrodo Red-loaded sperm before and during superfusion with CO_2/HCO_3^- (5%/25 mM).

Traces from four individual sperm cells. **(c)** Fluorescence change of pHrodo-Red loaded sperm during exposure to 25 mM HCO_3^- without additional CO_2 gassing. **(d)** PCF recording showing changes in pH_i (pHrodo-Red 12.5 µM) (F) and currents (I) upon superfusion of sperm with CO_2/HCO_3^- (5%/25 mM). Cell was held at V_m = -30 mV. The gap was caused by the software delay between the start of two subsequent recordings. **(e)** PCF recording from sperm of a wt mouse during superfusion with ES (black bar) and CO_2/HCO_3^- (blue bar). Changes in pH_i (upper) and currents (lower). V_m was first stepped from a holding potential of -30 mV to -90 mV to activate putative voltage-gated SLC9C1, followed by a step from -30 mV to +70 mV to activate H_v1. **(f)** Similar recordings from sperm of an *SLC9C1*$^{-/-}$ mouse.

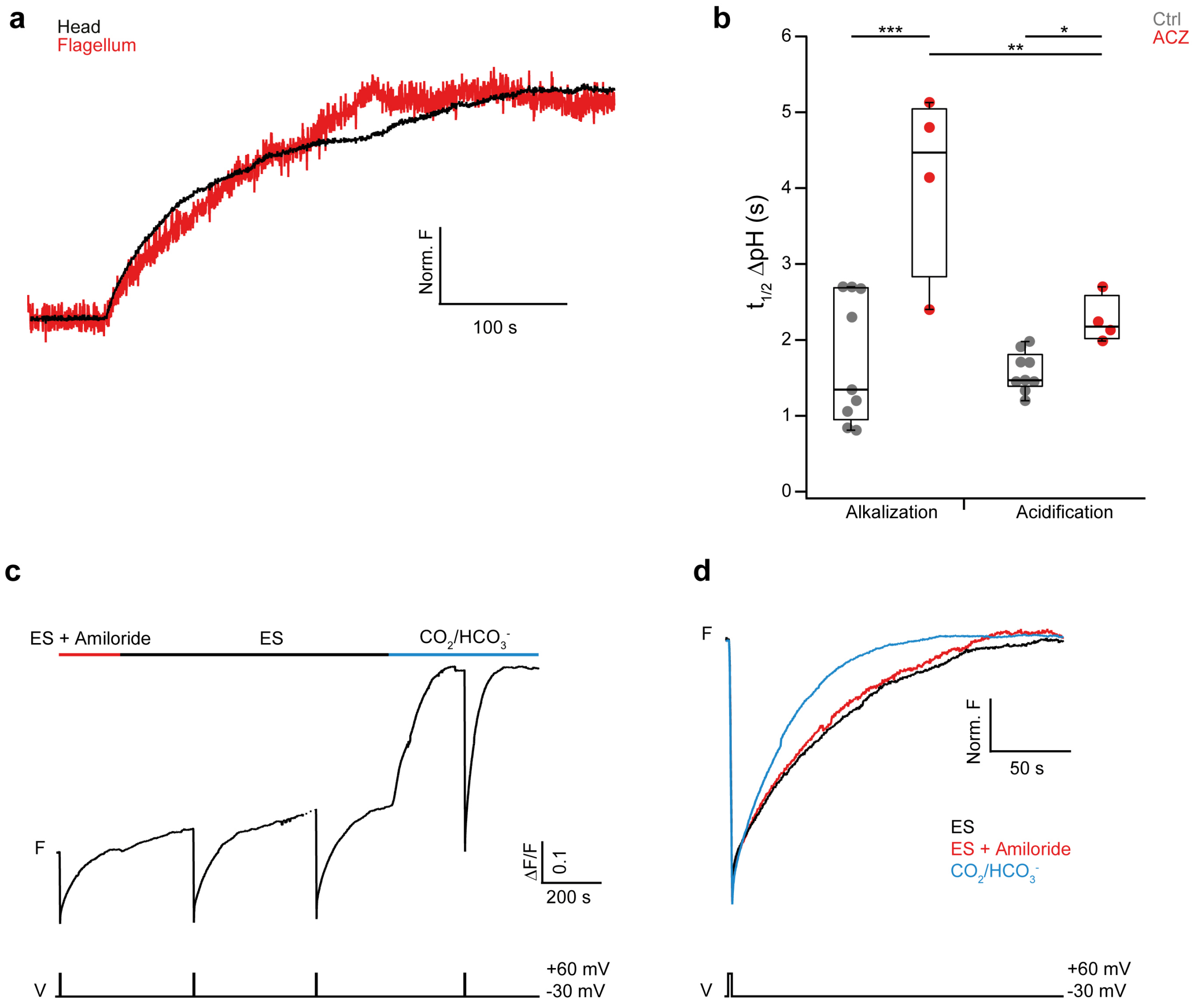

Supplementary Figure 5. Additional PCF experiments on human sperm and CHO cells expressing hH_v1.

(a) Time course of fluorescent change during solution exchange between pipette and sperm head (black) and flagellum (red) measured by PCF after break-in. The pipette was filled with 12.5 µM of pHrodo Red-maleimide. The holding potential V_m was -30 mV. **(b)** Half times $t_{1/2}$ of H_v1-mediated alkalization and subsequent acidification of a single sperm cell in absence (grey) and in presence of ACZ (100 µM, red; $n_{control} = 9$; $n_{ACZ} = 4$). Two-factor ANOVA followed by post-hoc Tukey's test for multiple comparison: $Ctrl_{Alk}$ vs. ACZ_{Alk} $p < 0.0001$; $Ctrl_{Acid}$ vs. ACZ_{Acid} $p = 0.4$; $Ctrl_{Alk}$ vs. $Ctrl_{Acid}$ $p = 0.9$; ACZ_{Alk} vs. ACZ_{acid} $p = 0.006$. **(c)** Changes in pH_i recorded by patch-clamp fluorometry in a CHO cell expressing hH_v1. The cell

was sequentially superfused with ES + amiloride, ES, and finally ES + CO_2/HCO_3^- (5%/25 mM). The pH_i response was elicited by stepping V_m from -30 mV to +60 mV. **(d)** Superposition of the normalized pH_i response in ES (black), ES + amiloride (500 µM) (red), and ES + CO_2/HCO_3^- (blue). Box plots represent 25%, 50% (median), and 75% quartiles, with whiskers displaying minimum and maximum data points. Source data is provided as a source data file.

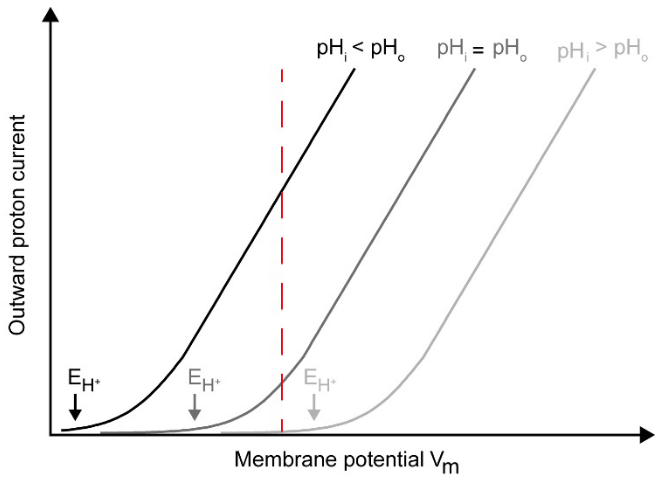

Supplementary Figure 6. Control of H_v1 activity by V_m and ΔpH_{tm} across the membrane.

Scheme illustrating the control of H_v1 by ΔpH_{tm} across the membrane and V_m. The interrupted line (red) illustrates that proton currents via H_v1 can be activated without a change in V_m.

Supplementary Table 1. Summary of proteomic studies.

Author	Method	Preparation	Detected proteins
Wang et al., 2013	LC-MS/MS	60% Percoll gradient centrifugation	4675 sperm proteins; 227 testis-specific
Baker et al., 2013	LC-MS/MS	15-75% Percoll gradient fractionation + head/tail separation by sonification & Percoll centrifugation	1429
Gu et al., 2011	LC-MS/MS	Swim-up	1019
Amaral et al., 2014	LC-MS/MS	50% Percoll centrifugation + leucocyte removal with Dynabeads + tail isolation by sonification & sucrose gradient centrifugation	1049

Supplementary Table 2: Signaling proteins identified in human sperm by MS

**genes**	**encoded proteins**	**Uniprot entry**	**Wang et al.**	**B1**	**B2**	**B3**
ABHD2	Monoacylglycerol lipase	P08910	+	+	+	+
ADCY10	Soluble adenylate cyclase	Q96PN6	+	+	+	+
AKAP3	A-Kinase anchor protein 3	O75969	+	+	+	+
AKAP4	A-Kinase anchor protein 4	Q5JQC9	+	+	+	+
ATP1A4	Na^+/K^+-ATPase alpha-4	Q13733	+	+	+	+
ATP2B1	Plasma membrane Ca^{2+}-transporting ATPase	P20020	+	+	+	+
ATP2B3	Plasma membrane Ca^{2+}-transporting ATPase	Q16720	-	+	+	+
ATP2B4	Plasma membrane Ca^{2+}-transporting ATPase	P23634	+	+	+	+
CA 2	Carbonic anhydrase 2	P00918	+	+	+	+
CA 4	Carbonic anhydrase 4	P22748	+	+	+	+
CATSPER 1	Sperm-specific Ca^{2+} channel 1	Q8NEC5	+	+	-	+
CATSPER 2	Sperm-specific Ca^{2+} channel 2	Q96P56	+	+	+	+
CATSPER 3	Sperm-specific Ca^{2+} channel 3	Q86XQ3	-	+	+	+
CATSPER 4	Sperm-specific Ca^{2+} channel 4	Q7RTX7	+	+	+	+
CATSPER B	Sperm-specific Ca^{2+} channel beta	Q9H7T0	+	+	+	+
CATSPER G	Sperm-specific Ca^{2+} channel gamma	Q6ZRH7	+	+	+	+
CATSPER D	Sperm-specific Ca^{2+} channel delta	Q86XM0	+	+	+	+
CATSPER E	Sperm-specific Ca^{2+} channel epsilon	Q5SY80	+	+	+	+
CATSPER Z	Sperm-specific Ca^{2+} channel zeta	Q9NTU4	+	+	-	-
EFCAB9	Ca^{2+}-binding protein	A8MZ26	+	+	+	+
HVCN1	H_v1 channel	Q96D96	+	+	+	+
KCNU1	Slo3 K^+ channel	A8MYU2	+	+	+	+
LRRC52	Auxiliary subunit of Slo3	Q8N7C0	+	+	+	+
PDE10A	cAMP-specific phoshodiesterase	Q9Y233	+	+	+	+
PPP1CC	Sperm-specific phosphatase	P36873		+	+	+
PRKACA	cAMP-dependent protein kinase catalytic subunit alpha	P17612	+	+	+	+
PRKAR1A	cAMP-dependent protein kinase type I regulatory subunit alpha	P10644	+	+	+	+
PRKAR2A	cAMP-dependent protein kinase type II regulatory subunit alpha	P13861	+	+	+	+
PRKAR1B	cAMP-dependent protein kinase type I regulatory subunit beta	P31321	+	+	+	+
SLC9C1	Na^+/H^+ exchange	Q4G0N8		+	-	+
SLC9C2	Na^+/H^+ exchange	Q5TAH2	-[1]	+	+	+
SLC9B1	Na^+/H^+ exchange	Q4ZJI4	-	+	+	+

SLC9B2	Na^+/H^+ exchange	Q86UD5	-	+	+	+
SLC26A3	Cl^-/HCO_3^- transport	P40879	-	+	+	+
SLC26A8	Cl^-, oxalate, SO_4^{2-} transport	Q96RN1	+	+	+	+
SLC30A1	Zn^{2+} transport	Q9Y6M5	+	+	+	+
SLC30A9	Zn^{2+} transport	Q6PML9	-	+	+	+
SLCO6A1	organic anion transport	Q86UG4	+	+	+	+
TMEM249	CatSper auxiliary subunit	Q2WGJ8	+	+	+	+

[1]Two peptides were detected in 1 from 3 measurements. The peptide sequences do not completely agree with the SLC9C1 sequence (86%). Probably SLC9C2 was detected.

**Supplementary Table 3:** Diverse other sperm-specific proteins identified in human sperm by MS						
**genes**	**encoded proteins**	**Uniprot entry**	**Wang et al.**	**B1**	**B2**	**B3**
ACR	Acrosin	P10323	+	+	+	+
CRISP1	Cystein-rich secretory protein 1	P54107	+	+	+	+
DNAH17	Dynein heavy chain 17 axonemal	Q9UFH2	+	+	+	+
GAPDH S	Sperm-specific glyceraldehyde-3-phosphate dehydrogenase	O14556	+	+	+	+
IZUMO1	Izumo sperm-egg fusion protein 1	Q8IYV9	+	+	+	+
IZUMO2	Izumo sperm-egg fusion protein 2	Q6UXV1	+	+	+	+
IZUMO3	Izumo sperm-egg fusion protein 3	Q5VZ72	+	+	+	+
IZUMO4	Izumo sperm-egg fusion protein 4	Q1ZYL8	+	+	+	+
LDHC	L-Lactate dehydrogenase c	P07864	+	+	+	+
ODF1	Outer dense fiber protein 1	Q14990	+	+	+	+
ODF2	Outer dense fiber protein 2	Q5BJF6	+	+	+	+
ODF3	Outer dense fiber protein 3A	Q96PU9	+	+	+	+
ODF3B	Outer dense fiber protein 3B	A8MYP8	+	+	+	+
PPP3R2	Calcineurin subunit B type 2	Q96LZ3	+	+	+	+
PLCZ1	Phospholipase C zeta 1	Q86YW0	+	+	+	+
SPACA1	Sperm acrosome-associated protein 1	Q9HBV2	+	+	+	+
SPACA3	Sperm acrosome-associated protein 3	Q8IXA5	+	+	+	+
SPACA4	Sperm acrosome-associated protein 4	Q8TDM5	+	+	+	+
SPACA5	Sperm acrosome-associated protein 5	Q96QH8	+	+	+	+
SPACA6	Sperm acrosome-associated protein 6	W5XKT8	-	+	+	+
SPACA7	Sperm acrosome-associated protein 7	Q96KW9	+	+	+	+
SPACA9	Sperm acrosome-associated protein 9	Q96E40	-	+	+	+

**Supplementary Table 4:** Signaling proteins that were *not* detected in human sperm by MS			
**genes**	**encoded proteins**	**Uniprot entry**	**Wang et al.**
CFTR	Cystic fibrosis transmembrane conductance	P13569	-
KCNMA1	K^+ channel Slo1	Q12791	-
KCNMB1	K^+ channel Slo1 beta subunit 1	Q16558	-
KCNMB2	K^+ channel Slo1 beta subunit 2	Q9Y691	-
KCNMB3	K^+ channel Slo1 beta subunit 3	Q9NPA1	-
KCNMB4	K^+ channel Slo1 beta subunit 4	Q86W47	-
LRRC26	K^+ channel Slo1 gamma subunit 1	Q2I0M4	-
LRRC38	K^+ channel Slo1 gamma subunit 4	Q5VT99	-
LRRC55	K^+ channel Slo1 gamma subunit 3	Q6ZSA7	-
SCNN1A	Na^+ channel ENaC subunit A	P37088	-
SCNN1B	Na^+ channel ENaC subunit B	P51168	-
SCNN1G	Na^+ channel ENaC subunit G	P51170	-
SLC4A1	Cl^-/HCO_3^- transport	P02730	+[1]
SLC4A2	Cl^-/HCO_3^- transport	P04920	-
SLC4A3	Cl^-/HCO_3^- transport	P48751	-
SLC4A4	Na^+/HCO_3^- co-transport	Q9Y6R1	-
SLC4A5	Na^+/HCO_3^- co-transport	Q9BY07	-
SLC4A7	Na^+/HCO_3^- co-transport	Q9Y6M7	-
SLC4A8	Na^+/HCO_3^- co-transport	Q2Y0W8	-
SLC4A9	anion exchange; protein 4	Q96Q91	-
SLC4A10	Na^+-driven Cl^-/HCO_3^- transport	Q6U841	-
SLC4A11	Na^+/HCO_3^- transporter-like	Q8NBS3	-
SLC8A1	Na^+/Ca^{2+} exchange 1	P32418	-
SLC8A2	Na^+/Ca^{2+} exchange 2	Q9UPR5	-
SLC8A3	Na^+/Ca^{2+} exchange 3	P57103	-
SLC22A14	organic cation transport	Q9Y267	-
SLC24A2	Na^+/Ca^{2+}-K^+ exchange	Q94I40	-
SLC24A4	Na^+/Ca^{2+}-K^+ exchange	Q8NFF2	-
SLC26A1	SO_4^{2-} transport	Q9H2B4	-
SLC26A2	SO_4^{2-} transport	P50443	-
SLC26A4	Na^+-independent Cl^-/I^- transport	Q43511	-
SLC26A5	prestin	P58743	-
SLC26A6	organic/inorganic anion transport	Q9BXS9	-
SLC26A7	Na^+-independent anion HCO_3^- transport	Q8TE54	-
SLC26A9	Cl^-/HCO_3^- exchange; HCO_3^- transport	Q7LBE3	-
SLC26A10	Cl^-/HCO_3^- exchange	Q8NG04	-
SLC26A11	Na^+-independent SO_4^{2-} transport	Q86WA9	-

[1]SLC4A1 is a house-keeping HCO_3^- transporter that is abundantly expressed in many cell types. A positive result may be due to small contaminations.

**Supplementary Table 5**: List of standard peptides	
**Protein**	**Standard peptide sequence**
Carbonic anhydrase 2	EPISVSSEQVLK
	QSPVDIDTHTAK
	VVDVLDSIK
Chloride anion exchanger	LIDAVGFSPLR
	YEFFDGEVK
Cystic fibrosis transmembrane conductance regulator	AYFLQTSQQLK
	SPFTHLVTSLK
Amiloride-sensitive sodium channel subunit alpha	KPCSVTSYQLSAGYSR
	YSSFTTLVAGSR
Electrogenic sodium bicarbonate cotransporter 1	SPTFLER
	FLFILLGPK
Solute carrier family 26A, member 6	CGVDVDFLISQK
	DTQALLSATQAMDLR
CatSperB	GFPQENEIIK
	FEASGPPTAFGNSR
CatSper1	SLIHDAPGPAASR
	SYGEDYHDELQR
CatSper2	LQYNLEER
CatSper3	LIQYSQGIR
	TVASVLLR
CatSper4	FNQEQESEVLNR
	VHDSSSQILLK
Potassium channel subfamily U member 1	QHITVPSVK
	YTSSYEALK

Supplementary Table 6. Summary of studies that examined changes in pH_i under capacitating conditions[1]

Authors	Species	Agent (mM)/system[2]	ΔpH	pH indicator
Meizel und Deamer (1978)	Hamster	none	acrosome	9-AA[3] ^{13}C-methylamine
Babcock et al. (1983)	Bovine	none	n.d.	CF[4]
Parrish et al. (1989)	Bovine	Heparin	0.4	CF[4]
Vredenburg-Willberg und Parrish (1995)	Bovine	Heparin	0.22[5]	BCECF[6]
Zeng et al. (1996)	Mouse	HCO_3^- (22.5)/o.s.	0.19[7]	BCECF
Nakanishi et al. (2001)	Mouse	HCO_3^- (25)/o.s.	acrosome	EGFP[8]
Demarco et al. (2003)	Mouse	HCO_3^- (5)/o.s.	0.15	BCECF
Xu et al. (2007)	Mouse	HCO_3^- (25)[9]	0.75	BCECF
Carlson et al. (2007)	Mouse	HCO_3^- (15)/o.s.	no change	BCECF
Cross and Razy-Faulkner (1997)	Human	HCO_3^- (25)/o.s.	0.14	BCECF
Lopez-Gonzalez et al. (2014)	Human	HCO_3^- (15)/o.s.	n.d.[10]	BCECF

[1]Studies that are cited in the literature as showing changes in pH_i under capacitating conditions; in fact, several of these studies do not use capacitating conditions involving HCO_3^-. [2]o.s., open system; [3]9-AA, 9-aminoacridine; [4]CF, carboxyfluorescein; [5]pH_i was constant for 3 h and then increased within 2 h; [6]BCECF, 2'-7'-bis(carboxyethyl)-5(6)-carboxyfluorescein, [7]capacitation was completed in < 2 h; [8]EGFP, pH-sensitive enhanced green fluorescent protein; [9]conditions were not clear from description, [10]ΔpH_i was not determined; only a subpopulation of sperm displayed a shift in flow cytometry.
