# Supplementary material for: Control of intracellular pH and bicarbonate by CO2 diffusion into human sperm
Source: Nat Commun. 2023 Sep 5;14:5395. doi: 10.1038/s41467-023-40855-0 (PMC10480191; doi:10.1038/s41467-023-40855-0)
Supplement: Supplementary file 3 — Description of Additional Supplementary Files [file 41467_2023_40855_MOESM3_ESM.pdf]

Description of Additional Supplementary Files

File Name: Supplementary Data 1
Description: Proteome from sperm of three different donors.

File Name: Supplementary Data 2
Description: MS data for all LOD values.
