# Supplementary material for: Control of intracellular pH and bicarbonate by CO2 diffusion into human sperm
Source: Nat Commun. 2023 Sep 5;14:5395. doi: 10.1038/s41467-023-40855-0 (PMC10480191; doi:10.1038/s41467-023-40855-0)
Supplement: Supplementary file 6 — Reporting Summary [file 41467_2023_40855_MOESM6_ESM.pdf]

nature portfolio

U. Benjamin Kaupp

Thomas Berger

Corresponding author(s): Henning Urlaub

Last updated by author(s): Jun 28, 2023

Reporting Summary

Nature Portfolio wishes to improve the reproducibility of the work that we publish. This form provides structure for consistency and transparency in reporting. For further information on Nature Portfolio policies, see our Editorial Policies and the Editorial Policy Checklist.

Statistics

For all statistical analyses, confirm that the following items are present in the figure legend, table legend, main text, or Methods section.

n/a	Confirmed	
☐	☒	The exact sample size (n) for each experimental group/condition, given as a discrete number and unit of measurement
☐	☒	A statement on whether measurements were taken from distinct samples or whether the same sample was measured repeatedly
☐	☒	The statistical test(s) used AND whether they are one- or two-sided<br>*Only common tests should be described solely by name; describe more complex techniques in the Methods section.*
☒	☐	A description of all covariates tested
☒	☐	A description of any assumptions or corrections, such as tests of normality and adjustment for multiple comparisons
☐	☒	A full description of the statistical parameters including central tendency (e.g. means) or other basic estimates (e.g. regression coefficient) AND variation (e.g. standard deviation) or associated estimates of uncertainty (e.g. confidence intervals)
☐	☒	For null hypothesis testing, the test statistic (e.g. F, t, r) with confidence intervals, effect sizes, degrees of freedom and P value noted<br>*Give P values as exact values whenever suitable.*
☒	☐	For Bayesian analysis, information on the choice of priors and Markov chain Monte Carlo settings
☒	☐	For hierarchical and complex designs, identification of the appropriate level for tests and full reporting of outcomes
☒	☐	Estimates of effect sizes (e.g. Cohen's d, Pearson's r), indicating how they were calculated

Our web collection on statistics for biologists contains articles on many of the points above.

Software and code

Policy information about availability of computer code

Data collection	Mass spectrometers were operated using the software Thermo XCalibur 4.4.16.14 for the Orbitrap Exploris™480 mass spectrometer and Thermo XCalibur 4.0.27.19 for the Q Exactive HF-X Hybrid Quadrupole-Orbitrap Mass Spectrometer. For aquisition of electrophysiology and fluorometry data ClampEx (version 10.2.0.15) and Andor Solis (version 4.30.30034.0) were used.
Data analysis	MaxQuant (version 1.6.5.0) software, Perseus (version 1.6.2) software, Skyline software (version 21.1.0.278), Microsoft Excel (version 2019) and the programming language R (version 4.2.2) as well as R Studio (2022.07.2) were used in analysing Mass Spectrometry data. For electrophysiology and fluorometry data ClampFit (10.0.3.03), Igor Pro (version 6.3.7.2) and ImageJ (version 1.52p) was used. For statistical analysis, GraphPad Prism 9 (Version 9.5.1) was used.

For manuscripts utilizing custom algorithms or software that are central to the research but not yet described in published literature, software must be made available to editors and reviewers. We strongly encourage code deposition in a community repository (e.g. GitHub). See the Nature Portfolio guidelines for submitting code & software for further information.

Data

Policy information about availability of data

All manuscripts must include a data availability statement. This statement should provide the following information, where applicable:

- Accession codes, unique identifiers, or web links for publicly available datasets
- A description of any restrictions on data availability
- For clinical datasets or third party data, please ensure that the statement adheres to our policy

The mass spectrometry proteomics data (Supplementary Table S2 and S8) has been deposited in the ProteomeXchange Consortium via the PRIDE partner repository with the dataset identifier PXD036819. Additionally, the Skyline analysis files have been deposited inPanorama (Data License: CC BY 4.0 und doi: 10.6069/6m29-at05). The UniProt complete Homo sapiens proteome sequence data base was used. All other data is available in the article or the source data that is provided as a Source Data file with this paper. All other data are available on request.

Human research participants

Policy information about studies involving human research participants and Sex and Gender in Research.

Reporting on sex and gender	male
Population characteristics	Human semen samples were donated by healthy adult caucasian males (age: 21 – 56 years).
Recruitment	Participants were recruited from the institute (caesar) and the University of Bonn. However, we are not aware of any bias that might affect the quality or properties of sperm.
Ethics oversight	The use of human sperm samples was approved by the ethics committee University of Bonn (042/17).

Note that full information on the approval of the study protocol must also be provided in the manuscript.

Field-specific reporting

Please select the one below that is the best fit for your research. If you are not sure, read the appropriate sections before making your selection.

☒ Life sciences ☐ Behavioural & social sciences ☐ Ecological, evolutionary & environmental sciences

For a reference copy of the document with all sections, see nature.com/documents/nr-reporting-summary-flat.pdf

Life sciences study design

All studies must disclose on these points even when the disclosure is negative.

Sample size	No sample size calculation was performed. The sample sizes were determined by the availability of the donors. For the mass spectrometry data, we performed three biological replicates to identify the human sperm proteome. The Venn diagramme (Fig. 2a) shows the high protein overlap between the replicates, supporting the notion that those three replicates provide a high protein coverage. Accordingly, subsequent quantitative MS experiments used 3-4 biological replicates.
Data exclusions	Mass Spectrometry measurements of low quality due to technical reasons were excluded from data analysis (for example, when the retention time windows did not cover the whole peak width or if the sensitivity of the machine was not up to the expected standard and, thereby, influencing accuracy of measurement). For electrophysiology, recordings with large leak currents that indicate inferior pipet/cell contact were excluded. Experiments often involved a solution switch in the perfusion system. Once in a while, mechanical instabilities (air, bubbles) cause optical and electrical artefacts. These experiments were excluded from further analysis.
Replication	For the mass spectrometry data, we performed 3-4 biological replicates for each set of experiments; all attempts with technically clean progression were successful. For the physiology measurements different numbers of samples were used; all technically successful experiments are reported and included in the analysis, and the numbers of all experiments and cells are specified in the manuscript.
Randomization	Randomization is not applicable. Comparisons are performed on different cell types or using different chemicals in separate (non-consecutive) experiments.
Blinding	The study was not blinded. Blinding is not common practice and neither useful nor practical in the context of a proteomics study and due to the technical complexity and exploratory nature of the techniques (patch-clamp electrophysiology, single sperm cell imaging) used.

Reporting for specific materials, systems and methods

We require information from authors about some types of materials, experimental systems and methods used in many studies. Here, indicate whether each material, system or method listed is relevant to your study. If you are not sure if a list item applies to your research, read the appropriate section before selecting a response.

Materials & experimental systems

n/a	Involved in the study
☒	☐ Antibodies
☐	☒ Eukaryotic cell lines
☒	☐ Palaeontology and archaeology
☐	☒ Animals and other organisms
☒	☐ Clinical data
☒	☐ Dual use research of concern

Methods

n/a	Involved in the study
☒	☐ ChIP-seq
☒	☐ Flow cytometry
☒	☐ MRI-based neuroimaging

Eukaryotic cell lines

Policy information about cell lines and Sex and Gender in Research

Cell line source(s)	HEK293 (ATCC-CRL-1573), Homo sapiens; CHO-K1 (ATCC-CCL-61), Cricetulus griseus; CHO-hHv1, CHO-K1 line stable expressing hHv1, produced by Dr. H. Körschen; human sperm, donated by male donors; mouse sperm (C57Bl/6N WT and SLC9C1-KO mice)
Authentication	None.
Mycoplasma contamination	Cell lines tested negative.
Commonly misidentified lines (See ICLAC register)	No commonly misidentified lines were used in this study.

Animals and other research organisms

Policy information about studies involving animals; ARRIVE guidelines recommended for reporting animal research, and Sex and Gender in Research

Laboratory animals	C57Bl/6N WT and SLC9C1-KO (B6; 129S6-Slc9a10tm1Gar/J, stock number: 007661, Jackson Laboratory); 2-5 months old. Mice were housed at 21°C with 55% relative humidity at a 12h light/dark cycle.
Wild animals	No wild animal have been used in this study.
Reporting on sex	male
Field-collected samples	No field-collected samples were used in this study.
Ethics oversight	Animal experiments were in accordance with the relevant guidelines and regulations and approved by the local authorities (Landesamt für Natur, Umwelt und Verbraucherschutz Nordrhein-Westfalen, LANUV).

Note that full information on the approval of the study protocol must also be provided in the manuscript.
